# Recent Advances in Drug-Induced Hypersensitivity Syndrome/Drug Reaction with Eosinophilia and Systemic Symptoms

**DOI:** 10.1155/2018/5163129

**Published:** 2018-03-18

**Authors:** Hideaki Watanabe

**Affiliations:** Department of Dermatology, Showa University School of Medicine, Tokyo, Japan

## Abstract

Drug-induced hypersensitivity syndrome (DIHS), also termed as drug reaction with eosinophilia and systemic symptoms (DRESS), is a multiorgan systemic reaction characterized by a close relationship with the reactivation of herpes virus. Published data has demonstrated that among patients with DIHS/DRESS, 75–95% have leukocytosis, 18.2–90% show atypical lymphocytes, 52–95% have eosinophilia, and 75–100% have hepatic abnormalities. Histologically, eosinophils were observed less frequently than we expected (20%). The mainstay of DIHS/DRESS treatment is a moderate dose of systemic corticosteroids, followed by gradual dose reduction. In this review, we will emphasize that elevations in the levels of several cytokines/chemokines, including tumor necrosis factor- (TNF-) *α* and the thymus and activation-regulated chemokine (TARC/CCL17), during the early stage of disease, are good markers allowing the early recognition of HHV-6 reactivation. TNF-*α* and TARC levels also reflect therapeutic responses and may be useful markers of the DIHS disease process. Recently, the pathogenic mechanism of T-cell activation triggered by human leukocyte antigen- (HLA-) restricted presentation of a drug or metabolites was elucidated. Additionally, we recently reported that dapsone would fit within the unique subpocket of the antigen-recognition site of HLA-B^∗^13:01. Further studies will render it possible to choose better strategies for DIHS prevention and therapy.

## 1. Introduction

Drug-induced hypersensitivity syndrome (DIHS), also termed drug reaction with eosinophilia and systemic symptoms (DRESS), is a multiorgan systemic reaction characterized by rashes, fever, lymphadenopathy, leukocytosis with eosinophilia and atypical lymphocytes, and liver dysfunction [1–4]. DIHS/DRESS is closely associated with the reactivation of herpes viruses, especially human herpesvirus 6 (HHV-6) and cytomegalovirus (CMV), in patients on long-term drug therapy [1–4]. DIHS/DRESS tends to exhibit a relatively later onset (≥2–8 weeks after commencing administration of the causative drug) than other types of drug eruptions. DIHS/DRESS is usually associated with only a limited number of drugs, including carbamazepine, phenytoin, phenobarbital, lamotrigine, dapsone, mexiletine, salazosulfapyridine, allopurinol, and minocycline [1–4]. Published works and our investigations indicated that oxidative metabolites of trichloroethylene, which may include trichloroacetylated protein adducts, can also induce a hypersensitivity syndrome quite similar to DIHS/DRESS [5]. The estimated risk at the first or second prescription of an aromatic antiepileptic drug is 2.3–4.5 in 10,000 [6]. This review explains the catachrestic features of DIHS/DRESS, the markers allowing early recognition of HHV-6 reactivation, and the recent advances in the genetics of DIHS/DRESS.

## 2. Criteria for DIHS/DRESS

DRESS, first defined in 1996 by Bocquet et al. [2], presents with a constellation of symptoms and signs, the main features being a cutaneous eruption after exposure to the culprit drug, associated with fever and organ involvement (Table 1(a)). Hematologic (lymphadenopathy, eosinophilia, and atypical lymphocytosis) and hepatic (elevation of serum transaminases) manifestations are frequently reported [2]. Subsequently, inclusion criteria for HSS/DRESS were defined in RegiSCAR, a research group investigating severe cutaneous adverse reactions (SCAR), and a scoring system for classifying DRESS cases was established (Table 1(b)) [7]. In 2006, a Japanese consensus group established a set of criteria for the diagnosis of DIHS (Table 1(c)) [3]. The diagnosis of the typical syndrome requires all seven criteria. Importantly, a series of >60 patients diagnosed by clinical findings consistently showed detection of HHV-6 reactivation in the vast majority of patients who satisfied the other six criteria and showed clinical manifestations consistent with those reported by Bocquet et al. [2], but not in those with other types of drug eruption such as papillomacular rash, Stevens–Johnson syndrome (SJS), and toxic epidermal necrolysis (TEN). In contrast, HHV-6 reactivation is rarely detected in patients with a tendency toward milder disease. Thus, it appears that patients fulfilling the criteria of DIHS may represent those with a more severe form of DRESS [3].

## 3. Clinical Findings

DIHS/DRESS commonly commences with a fever, followed soon by a maculopapular rash that is usually pruritic, and a variable degree of lymphadenopathy [1–4]. The rash often generalizes to become severe exfoliative dermatitis or erythroderma [1, 2]. Symptom onset is highly variable; usually, patients develop two or three symptoms followed by the stepwise development of other symptoms [1, 2]. In many severe cases, the symptoms continue to deteriorate, and/or several flare-ups occur, in the weeks after the offending drug is stopped [1–4].

**Figure 1:**
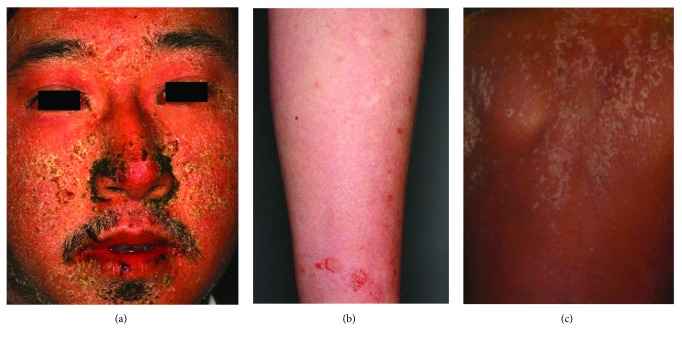
Clinical findings in patients with drug-induced hypersensitivity syndrome (DIHS). (a) Edema and erythema with scaling were observed on the face. Crusts were seen on the lateral surfaces of the nose and around the lips. (b) A diffuse erythematous rash and blisters were seen on the forearm. (c) Diffuse erythema with scaling on the trunk was consistent with erythroderma.

The skin manifestations of DIHS are maculopapular rash, erythema multiforme, exfoliative dermatitis, acute generalized exanthematous pustular dermatosis-like eruption, and erythroderma [1–4]. We recently reviewed 20 patients with DIHS/DRESS, including 7 with maculopapular rash type, 5 with EM type, and 8 with erythroderma [8]. Initially, the upper trunk, face, and upper extremities are affected, followed by the involvement of lower extremities. Periorbital, facial edema with erythema and numerous scales and crusts around the nose and lips are characteristic features of DIHS/DRESS at the early stage (Figure 1(a)) [1, 5]. In some cases, bullous lesions are found on the forearm, which are also characteristic features of DIHS/DRESS (Figure 1(b)) [5]. The rash often generalizes into severe exfoliative dermatitis or erythroderma (Figure 1(c)) [1, 2, 5]. There is usually no mucocutaneous involvement, which helps distinguish DIHS/DRESS from other forms of severe drug eruptions, such as SJS and TEN [1].

## 4. Laboratory Data

Leukocytosis with atypical lymphocytes and eosinophilia of varying degree is a prominent feature of the syndrome [1]. Leukocytosis was observed in 99 of 104 (95%) patients reported by the RegiSCAR study group [4] and 15 of 20 (75%) Japanese patients reported by us [8]. The presence of atypical lymphocytes was demonstrated in 68 of 102 (67%) cases reported by the RegiSCAR study group [4], 38 of 60 (63%) reported from Taiwan [9], 18.5% patients reported from Thailand [10], 4 of 22 (18.2%) reported from Singapore [11], and 18 of 20 (90%) Japanese cases reported by us [8]. Eosinophilia was observed in 108 of 114 (95%) cases reported by the RegiSCAR study group [4], 31 of 60 (52%) reported from Taiwan [9], 70.4% patients reported from Thailand [10], 22 out of 27 (81.5%) reported from Singapore [11], and 13 of 20 Japanese patients (65%) reported by us [8]. Eosinophilia may often be delayed for 1 to 2 weeks and may occur even after the elevations in liver enzyme levels return to baseline [1]. In DIHS/DRESS, elevation of liver enzyme levels, the most common finding related to internal organ involvement [1], was found in 86 of 114 (75%) cases reported by the RegiSCAR study group [4] and 26 of 27 (96.3%) reported by both Singapore and Thailand [10, 11]; 48 (80%) cases in Taiwan had levels double that of normal [9]. We reported that all 20 Japanese patients with DIHS/DRESS had hepatic abnormalities (alanine aminotransferase (ALT) above the normal range of 5–25 IU/L and 14 patients [70%] had a serum ALT > 100 IU/L) [8]. Renal involvement was found in 40 of 108 (37%) cases reported by the RegiSCAR study group [4], 24 of 60 (40%) reported from Taiwan [9], 4 of 27 (14.8%) reported from Singapore [11], and 7 of 20 (35%) Japanese patients reported by us [8]. It is well known that the frequency of renal involvement is higher in patients with DIHS due to allopurinol [1].

## 5. Histopathology of DIHS

It is crucial for the diagnosis of SJS/TEN to examine histopathological findings to determine whether apoptotic keratinocytes are scattered in the epidermis [12]. On the other hand, it is noteworthy that none of the criteria of DIHS/DRESS [2, 3, 7] rely on histopathology. Until recently, few examinations of histopathological findings of DIHS/DRESS were reported. Ortonne et al. [13] conducted a retrospective study on 50 skin biopsies from 36 patients with DIHS/DRESS and demonstrated that patients with DIHS/DRESS frequently show foci of interface dermatitis, involving cutaneous adnexa. Eosinophils were seen in only 20% and neutrophils in 42% of cases. Eczematous, interface dermatitis, and acute generalized exanthematous pustulosis-like and erythema multiforme-like patterns were observed in skin biopsy samples from patients with DIHS/DRESS. The association of two or three of these patterns in a single biopsy was significantly more frequent in DRESS than in a series of nondrug-induced dermatoses and appeared to be more marked in DRESS with severe cutaneous lesions than in DRESS with less severe lesions. Interestingly, higher proportions of CD8+ and granzyme B+ lymphocytes were observed in DRESS with severe cutaneous eruptions. Furthermore, FoxP3+ regulatory T cells were found within the skin infiltrates in the acute phase of DRESS; however, these cells were not numerous [13]. In addition, they found apoptotic keratinocytes in 60% of DRESS syndrome cases [13]. This observation was consistent with the report by Walsh et al. [14], which showed that the presence of apoptotic keratinocytes correlated with a more aggressive phenotype with liver injury and an erythema multiforme-like cutaneous condition. Chi et al. [15] also found that skin biopsies of DIHS/DRESS displayed various inflammatory aspects and showed that interface dermatitis with apoptotic keratinocytes was more frequent in DIHS/DRESS than in maculopapular rash.

## 6. Treatment

The mortality rate of DIHS has recently been estimated to be 2–14% [7, 9]. The mainstay of treatment is systemic corticosteroids [1]. Wei et al. reviewed 91 cases with DRESS in Taiwan [9]. Patients treated with systemic corticosteroids lived longer than those not treated with corticosteroids (average 36.3 versus 12.7 days). In the survival group, approximately three-quarters of the patients received systemic corticosteroids, but their resolution time was 8 days longer than those without. A study from Singapore demonstrated that 25 of 27 (92.6%) patients with DIHS/DRESS received systemic corticosteroids, with no deaths resulting from DIHS/DRESS during the follow-up period in their case series [11].

Systemic corticosteroids, recommended for most cases of DIHS/DRESS, should be initiated at a dose of 40–60 mg prednisone equivalent daily, followed by a gradual dose reduction of prednisone given over 10 weeks to prevent rapid reconstitution of valid immune responses against various pathogens; however, the mild form can resolve spontaneously over a period of weeks [1, 17]. The development of autoimmune diseases, such as lupus erythematosus and autoimmune thyroiditis, along with the generation of autoantibodies, was preferentially observed in the noncorticosteroid treatment group in the late phase (>6 months) of DIHS/DRESS [16, 17]. Severe liver damage and noncorticosteroid therapy during the acute stage were associated with the subsequent generation of autoantibodies against plakin family proteins [16]. Therefore, corticosteroids, especially if administered in the acute stage, may improve the long-term outcome [17]. Recently, Leman et al. [18] described the successful treatment of a case of DIHS/DRESS with a tumor necrosis factor- (TNF-) *α* inhibitor containing lithium carbonate. However, this is the only report of DIHS/DRESS treatment with a TNF-*α* inhibitor, and further clinical studies are required.

## 7. Biomarkers of Disease Severity and HHV-6 Reactivation in DIHS/DRESS

A major clinical focus during the diagnosis of DIHS and the selection of the most appropriate treatment is whether the reactivation of members of the Betaherpesvirinae subfamily, including HHV-6, develops subsequently to the drug hypersensitivity reaction [1–4]. HHV-6 DNA is detected in serum about 3–5 weeks after disease onset, followed by dramatic rises in anti-HHV-6 IgG titers [1, 17]. Shiohara et al. performed a sequential analysis of viral loads and found that the cascade of reactivation events initiated by HHV-6 or EBV extended, after some delay, to HHV-7 also and eventually to CMV [1]. In our previous study, when both HHV-6 and CMV became reactivated in the same DIHS patients, HHV-6 DNA was detected 21–35 days after disease onset and followed 10–21 days later by CMV DNA; the CMV IgG antibody titer also increased 10–21 days after elevation of the HHV-6 antibody titer [8]. In the cited study, 80% of DIHS patients exhibited HHV-6 reactivation [8]. The magnitudes of 2HHV-6 reactivation as evidenced by the increases in HHV-6 DNA levels correlated well with the severities of the inflammatory responses [1]. However, no useful predictive marker of HHV-6 reactivation has yet been widely accepted. Moreover, useful biomarkers of the DIHS disease process have not yet been reported.

### 7.1. Tumor Necrosis Factor-*α*

**Figure 2:**
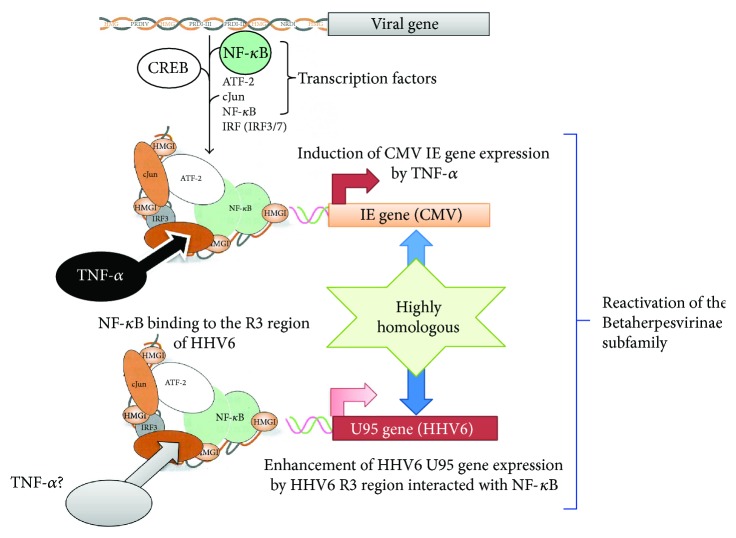
Possible involvement of tumor necrosis factor- (TNF-) *α* in the reactivation of cytomegalovirus (CMV) and human herpesvirus (HHV)-6. TNF-*α* may play a role in reactivation of Betaherpesvirinae subfamily members, including CMV and HHV-6. TNF-*α* enhances the expression of CMV immediate early gene products. Enhancement of HHV-6 U95 gene expression by the R3 region of HHV-6 might interact with nuclear factor- (NF-) *κ*B by TNF-*α*.

We recently conducted comparative assessments of, and detailed examinations on, patients with DIHS and measured their serum protein levels [8]. We found that the serum levels of TNF-*α* before treatment were significantly higher in the HHV-6 reactivation group than in the non-HHV-6 reactivation group. In that, a TNF-*α* level of 12 pg/mL allowed the detection of HHV-6 reactivation [8]. Increased levels of proinflammatory cytokines including TNF-*α* and IL-6 have been reported in patients with HHV-6 infections (severe cases of exanthema subitum) and CMV infections [19, 20]. However, the exact mechanisms of the reactivation of these viruses have not been fully elucidated. On the basis of both molecular and biological analyses, HHV-6, which is very similar to CMV, is the prototypic member of the Betaherpesvirinae [21, 22]. Numerous *in vitro* and *in vivo* studies have sought to elucidate the mechanisms of CMV reactivation and have reported that cytokine production, particularly of TNF-*α*, was implicated in reactivation [23–25]. TNF-*α* induces the expression of CMV immediate early (IE) gene products, potentially initiating viral replication from the latent state [26]. Expression of CMV IE genes is controlled by IE promoter/enhancer regions, which contain binding sites for NF-*κ*B, ATF (CREB), and Sp1. The NF-*κ*B and ATF (CREB) sites are critical in terms of the regulation of IE gene expression [26, 27]. In contrast, the R3 region of HHV-6 contains multiple putative binding sites for cellular transcription factors, including PEA3, NF-*κ*B, and AP-2. Via interactions with NF-*κ*B, this region strongly enhances the promoter activity of the U95 gene, a potential homolog of the murine CMV IE2 gene [21]. These observations and our finding that the serum levels of TNF-*α* were significantly higher in the HHV-6 reactivation group than in the non-HHV-6 reactivation group of DIHS patients suggest that TNF-*α* may play a crucial role in HHV-6 reactivation (Figure 2). Moreover, an increase in the level of TNF-*α* before the commencement of treatment may be an especially good biomarker allowing early recognition of HHV-6 reactivation in patients with DIHS. Consistent with this finding, it was reported that the TNF-*α* level was higher in hematopoietic stem cell transplantation recipients exhibiting HHV-6 reactivation than in those who did not exhibit reactivation. Kamijima et al. recently investigated 28 patients with trichloroethylene hypersensitivity syndrome and recorded the times of reaction onset after exposure to trichloroethylene/other drugs, the clinical manifestations, blood data, and the duration of virus reactivation [28]. It was found that an elevated TNF-*α* level on admission correlated significantly with an increase in HHV-6 DNA during the clinical course. This supports our suggestion that an increased level of TNF-*α* prior to the commencement of treatment may be an excellent biomarker allowing early recognition of HHV-6 reactivation in patients with DIHS [8]. Moreover, in our earlier study, the TNF-*α* levels decreased significantly in parallel with the responses to treatment only in the DIHS group. To date, no widely accepted biomarkers of the DIHS disease process are available. Yoshikawa et al. reported elevated levels of TNF-*α* and IL-6 levels in four of six DIHS patients at the time of disease onset [29], indicating that the serum level of this protein reflected DIHS development. However, this report included only a small number of DIHS/DRESS cases (*n* = 6), making it difficult to discuss or compare these results with ours.

### 7.2. Interferon-Induced Protein 10

C-X-C motif chemokine 10 (CXCL10), also known as interferon- (IFN-) *γ*-induced protein 10 kDa (IP-10), plays an important role in the recruitment of antiviral-specific cytotoxic T lymphocytes into the target tissue [30]. Serum and/or tissue expression of IP-10 is increased in organ-specific autoimmune diseases and in interface dermatitis [30]. Contrary to other reports [8, 29], Chen et al. [31] demonstrated that many proinflammatory cytokines and chemokines, including interleukin- (IL-) 1*β*, IL-2, IL-6, IFN-*γ*, and TNF-*α*, were significantly lower in DIHS/DRESS patients with HHV-6 reactivation when compared to those without HHV-6 reactivation. In addition, these mediators were significantly lower before and during HHV-6 reactivation, compared to cytokine levels after HHV-6 reactivation in the same patients [31]. These findings suggest the importance of the timing of sample collection and that the influence of systemic corticosteroids in patient treatment should be considered carefully. Future investigations using larger numbers of samples will be needed.

### 7.3. Thymus and Activation-Regulated Chemokine and Other Th2-Type Cytokines/Chemokines

Ogawa et al. recently reported that the serum thymus and activation-regulated chemokine (TARC) levels were markedly higher in patients with DIHS/DRESS than in patients with other forms of drug eruption including SJS/TEN and maculopapular erythema [32]. It was found that the serum TARC levels of patients in the acute stage of DIHS correlated with disease activity and that the serum TARC levels in patients exhibiting HHV-6 reactivation were significantly higher than those in patients not exhibiting HHV-6 reactivation [33]. Interestingly, the serum TARC levels correlated with the RegiSCAR group diagnostic score for DRESS [33]. Such findings led us to suggest a pathogenic link between serum TARC levels and HHV-6 reactivation. Although the precise mechanism remains largely unknown, one possible explanation is that immunosuppression triggers HHV-6 reactivation via regulatory T cell activation induced by elevated TARC levels. Another possibility is that elevated TARC levels directly activate HHV-6 via the chemokine receptor homologues of HHV-6 [33].

Yawalkar et al. [34] examined skin sections from patients with characteristic, acute, drug-induced, maculopapular exanthem to determine the potential role of IL-5 and distinct chemokines in the recruitment and activation of eosinophils into the skin. They demonstrated that drug-induced maculopapular exanthems express significantly increased amounts of IL-5 and eotaxin [34]. However, whether these Th2 cytokines/chemokines are involved in the reactivation of HHV-6 in DIHS/DRESS has not yet to be determined.

### 7.4. Plasmacytoid Dendritic Cells

Plasmacytoid dendritic cells (pDCs) play a defensive role against viruses [35]. Previously, we demonstrated that pDCs accumulate in the skin of patients with DIHS/DRESS and that the number of pDCs in circulation decreases significantly around the time of viral reactivation. Upon viral infection, stimulated pDCs are prompted to differentiate into DCs by autocrine IFN-*α* and TNF-*α* and to prime naive CD4+ T cells to produce IFN-*γ* and IL-10 [36]. In addition, pDCs preferentially secrete the proinflammatory chemokine macrophage inflammatory protein- (MIP-) 1*α*, which recruits mostly Th1-type effector cells and causes the production of other proinflammatory cytokines [37]. Therefore, decreased levels of proinflammatory cytokines/chemokines may result from decreased levels of pDCs and depress the antiviral capacity in patients with DRESS. After reactivation, HHV-6 may further modulate the release of these cytokines from peripheral blood mononuclear cells, including IFN-*γ*, TNF-*α*, and IL-1*β*, as reflected by their increased levels in the blood [31].

### 7.5. High-Mobility Group Box-1

Damage-associated molecular pattern molecules (DAMPs) released from damaged cells are signals for initiating immune responses in various organs through their activation after interacting with pattern recognition receptors and/or Toll-like receptors, thereby promoting rapid recruitment of bone marrow-derived leukocytes to the target tissues for inflammation and regeneration under various aseptic inflammatory conditions [38, 39]. High-mobility group box (HMGB)-1, one of the most well-known DAMP members, is a nonhistone protein with dual functions: intercellular transcriptional regulation by loose binding to chromatin and extracellular high potency signaling of inflammation to attract and activate various immunocompetent cells including monocytes and myeloid cells [39]. Hashizume et al. [40] demonstrated that the circulating monomyeloid precursors in patients with DIHS were mostly CD11b+ CD13+ CD14 CD16^high^ and showed substantial expression of skin-associated molecules, such as CCR4. CD13+ CD14 cells were also found in DIHS skin lesions, suggesting skin recruitment of this cell population. High levels of HMGB-1 were detected in blood and skin lesions in the active phase of patients with DIHS, and recombinant HMGB-1 showed functional chemoattractant activity for monocytes/monomyeloid precursors *in vitro*. HHV-6 infection of the skin-resident CD4+ T cells was confirmed by the presence of its genome and antigen. This infection was likely mediated by monomyeloid precursors recruited to the skin, as normal CD4+ T cells gained HHV-6 antigen after *in vitro* coculture with highly virus-loaded monomyeloid precursors from patients. Hashizume et al. [40] suggested that monomyeloid precursors harboring HHV-6 are navigated by HMGB-1 released from damaged skin and likely cause HHV-6 transmission to skin-infiltrating CD4+ T cells, which is an indispensable event for HHV-6 replication. Another group also reported increased HMGB-1 levels during the acute stage of DIHS [41]. However, contrary to those reports, Nakajima et al. showed that the serum level of HMGB-1 in SJS/TEN was higher than that of DIHS [42]. Further investigations are needed.

## 8. Pharmacogenomic Features of Severe Cutaneous Adverse Reactions Including DIHS/DRESS

To date, genetic factors have been shown to play important roles in several types of drug eruptions, including DIHS/DRESS. For example, the human leucocyte antigen- (HLA-) B^∗^15:02 allele was identified as an important predictor of risk for the development of both carbamazepine-induced SJS and TEN in a southeast Asian population [43]; in contrast, the HLA-A^∗^31:01 allele was found to be relevant in European [44] and Japanese populations [45]. Many other pharmacogenomic features of SCAR have been discovered, some of which are ethnically specific. For example, HLA-B^∗^57:01 is associated with abacavir hypersensitivity in Caucasians; HLA-B^∗^58:01 with allopurinol-SCAR (both SJS/TEN and DIHS) in Chinese, Japanese, Koreans, Thais, and Europeans; HLA-A^∗^31:01 with CBZ-SCAR (DIHS) in Han Chinese, Europeans, Japanese and Koreans; HLA-B^∗^15:02 with phenytoin-SJS/TEN in Han Chinese; and HLA-B^∗^B^∗^59:01 and CW^∗^01:02 with methazolamide-SJS/TEN in Koreans and Japanese (Table 2) [46].

The immunogenic complexes involved in T cell-mediated adverse drug reactions contain three components: an HLA protein, a peptide, and a drug [47]. To date, three principal models for this interaction have been developed, based on differences in the roles played by cellular metabolism and antigen processing [48–51]. These are the hapten/prohapten pharmacological interaction with an immune receptor model (the p.i. model) and the altered peptide repertoire model. Illing et al. recently suggested that abacavir hypersensitivity syndrome could be explained by reference to the altered peptide repertoire model [47, 48].

Recently, an HLA class I allele, HLA-B^∗^13:01, has been identified as a marker of susceptibility to DIHS attributable to dapsone (dapsone hypersensitivity syndrome) [52–54]. It was initially unclear how dapsone interacted with HLA-B^∗^13:01.

## 9. Computational Analyses of the Dapsone/HLA-B^∗^13:01 Interactions

**Figure 3:**
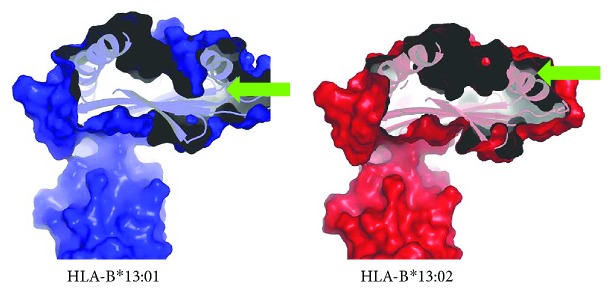
An extra-deep subpocket around the F-pocket of the antigen-binding site of HLA-B^∗^13:01, which was not observed in HLA-B^∗^13:02. HLA-B^∗^13:01 (blue) had an extra-deep subpocket (green arrows) absent from HLA-B^∗^13:02 (red).

**Figure 4:**
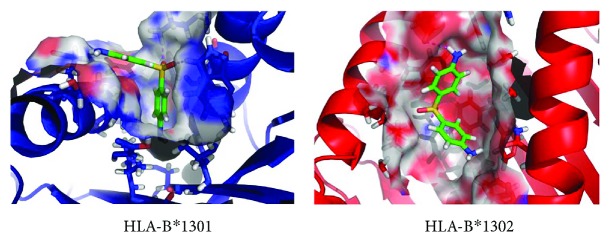
Dapsone binds more tightly to HLA-B^∗^13:01 than to 13:02. Binding models before molecular dynamic simulations for dapsone–HLA-B^∗^13:01 (blue) and dapsone–HLA-B^∗^13:02 (red), based on observations of the stick representation of their HLA three-dimensional homology models. Dapsone (green) inserts are deeper in HLA-B^∗^13:01 (blue) than in HLA-B^∗^13:02 (red).

It was most surprising that HLA-B^∗^13:01 exhibited a strong association with DIHS attributable to dapsone (dapsone hypersensitivity) but HLA-B^∗^13:02 did not. Only three amino acid residues of 338 differ between HLA-B^∗^13:01 and HLA-B^∗^13:02 [55]. These correspond to I^94^I^95^R^97^ in HLA-B^∗^13:01 and T^94^W^95^T^97^ in HLA-B^∗^13:02. When we compared the molecular surface representations of the antigen-binding sites, we found that HLA-B^∗^13:01 had an extra, and deep, subpocket around the F-pocket of the antigen-binding site, which was not present in HLA-B^∗^13:02 (Figure 3) [55]. The size of the extra subpocket seemed appropriate to accommodate the aniline group, suggesting that dapsone binds tightly to HLA-B^∗^13:01 using this unique subpocket (Figure 4). In fact, Illing et al. recently suggested that abacavir hypersensitivity syndrome could be explained by reference to the altered peptide repertoire model [47, 48]. In the altered peptide repertoire model, the drug interacts with the antigen-binding cleft of a specific HLA allele and alters the binding of self-peptides to the HLA molecule. This results in a T cell response. X-ray crystallography revealed that abacavir was specifically bound in the vicinity of the F-pocket of the antigen-binding cleft of the HLA-B^∗^57:01 allele. This region was identified as a marker of susceptibility to abacavir hypersensitivity syndrome. From these findings, an “altered peptide repertoire” model involving the binding of dapsone to HLA-B^∗^13:01 may also be appropriate analogous to the abacavir allergy model.

## 10. Conclusion

During the course of DIHS, HHV-6 reactivation triggers symptom recurrence and may be fatal by causing serious dysfunctions including liver failure. Therefore, it is essential to identify factors predictive of virus reactivation. In this review, we have emphasized that several cytokines/chemokines including levels of TNF-*α* and TARC are good biomarkers of virus reactivation; however, further investigations are required. Moreover, the association between causative drugs and genetic factors, including HLA polymorphisms, renders it possible to choose appropriate treatments and improve patient outcomes.

## Figures and Tables

**Table:** (a) Diagnostic criteria for drug reaction with eosinophilia and systemic symptoms (DRESS) [2].

Diagnosis of DRESS is confirmed by the presence of all of the following criteria:	
(1) Cutaneous drug eruption	
(2) Adenopathies ≥ 2 cm in diameter or hepatitis (liver transaminases ≥ 2 times upper limit of normal) or interstitial nephritis or interstitial pneumonitis or carditis	
(3) Hematologic abnormalities: eosinophilia ≥ 1.5 × 10^9^ L^−1^ or atypical lymphocytes	

**Table:** (b) Criteria for potential cases of drug reaction with DRESS by RegiSCAR [7].

(1) Hospitalization	
(2) Reaction suspected to be drug-related	
(3) Acute skin rash^∗^	
(4) Fever above 38°C^∗^	
(5) Enlarged lymph nodes in at least two sites^∗^	
(6) Involvement of at least one internal organ^∗^	
(7) Blood count abnormalities	
(i) Lymphocytes above or below the laboratory limits^∗^	
(ii) Eosinophils above the laboratory limits^∗^	
(iii) Platelets below the laboratory limits^∗^	

^∗^Three or more criteria required. RegiSCAR: research group investigating severe cutaneous adverse reactions (SCAR) [7].

**Table:** (c) Diagnostic criteria for drug-induced hypersensitivity syndrome (DIHS) established by a Japanese consensus group [3].

(1) Maculopapular rash developing 3 weeks after starting with a limited number of drugs	
(2) Prolonged clinical symptoms 2 weeks after discontinuation of the causative drug	
(3) Fever (≥38°C)	
(4) Liver abnormalities (alanine aminotransferase ≥ 100 U·L^−1^)^a^	
(5) Leukocyte abnormalities (at least one present)	
(a) Leukocytosis (≥11 × 10^9^ L^−1^)	
(b) Atypical lymphocytosis (≥5%)	
(c) Eosinophilia (≥1.5 × 10^9^ L^−1^)	
(6) Lymphadenopathy	
(7) Human herpesvirus 6 reactivation	

The diagnosis is confirmed by the presence of the seven criteria above (typical DIHS) or of the first five (1–5) criteria (atypical DIHS). ^a^This can be replaced by other organ involvement, such as renal involvement.

**Table 2:** Specific human leucocyte antigen (HLA) types and associated drugs in severe drug eruptions.

Associated drug	HLA allele	Ethnicity
Abacavir	B^∗^57:01	Caucasian, Thai, Cambodian
Allopurinol	B^∗^58:01	Han Chinese, Thai, Japanese, Korean
Carbamazepine	B^∗^15:02	Han Chinese, Thai, Indian, Malaysian
	B^∗^15:11	Japanese, Korean, Han Chinese
	B^∗^59:01	Japanese
	A^∗^31:01	Japanese, Han Chinese, European, Korean
Cold medicine	A^∗^02:06	Japanese, Korean
Dapsone	B^∗^13:01	Han Chinese, Thai
Methazolamide	B^∗^59:01	Korean, Japanese, Han Chinese
Nevirapine	DRB1^∗^01:01	Australian, French
	B^∗^14:02 (or Cw^∗^08:02)	European
	B^∗^35:05	Thai
	Cw^∗^08:01/Cw^∗^08:02	Sardinian, Japanese
Phenobarbital	HLA-A^∗^01:01	Thai
	HLA-B^∗^13:01	Thai
Phenytoin	B^∗^15:02	Han Chinese, Thai
	HLA-B^∗^13:01	Thai
	HLA-B^∗^56:02/04	Thai
Sulfamethoxazole	B^∗^38	European

This table is modified from [46].
